# Supplementary material for: Population and distribution of wild Asian elephants (Elephas maximus) in Phu Khieo Wildlife Sanctuary, Thailand
Source: PeerJ. 2021 Jul 29;9:e11896. doi: 10.7717/peerj.11896 (PMC8325913; doi:10.7717/peerj.11896)
Supplement: Supplemental Information 7 — - [file peerj-09-11896-s007.docx]

**Supplementary S1** Wild Asian elephant sex and age identifications

**1. Age Identification**

The age of wild Asian elephants can be classified into four: 1) calf, 2) juvenile, 3) sub-adult and 4) adult (Arivazhagan and Sukumar, 2008) (Table 1 and Fig. 1).

**Table 1** Rule of age and height for the wild elephant

| **Major age classes** | **Age (year)** | **Height (feet)** | |
| --- | --- | --- | --- |
|  |  | **Male** | **Female** |
| Calf | 0-1 | 3-4 |  |
| Juvenile | 1-5 | 4-6 |  |
| Sub-adult | 5-15 | 6-8 | 5-7 |
| Adult | >15 | >8 | >7 |

Source: Arivazhagan and Sukumar (2008)


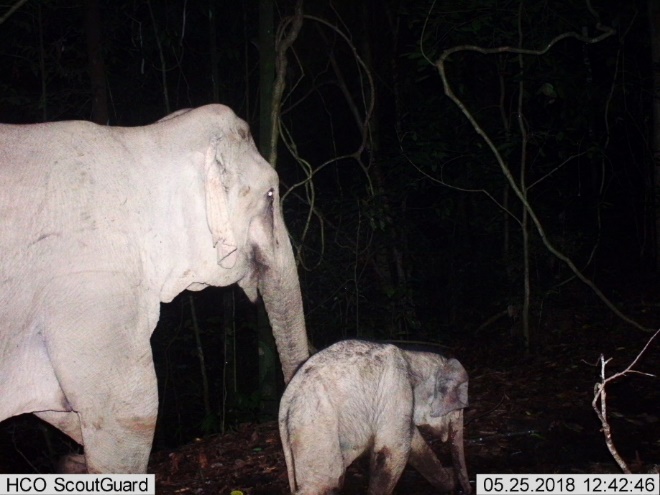

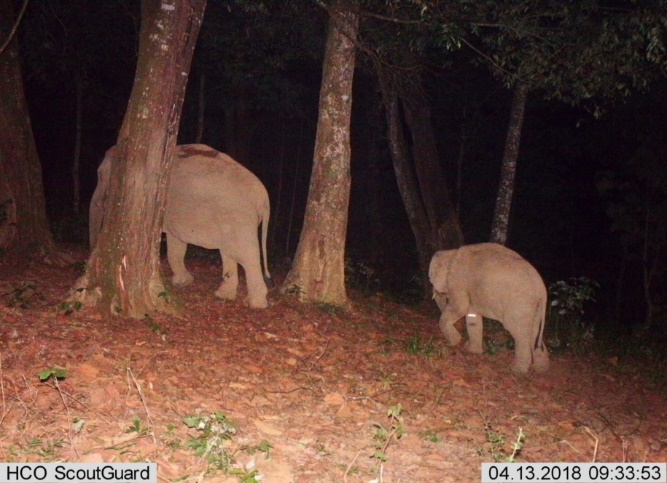

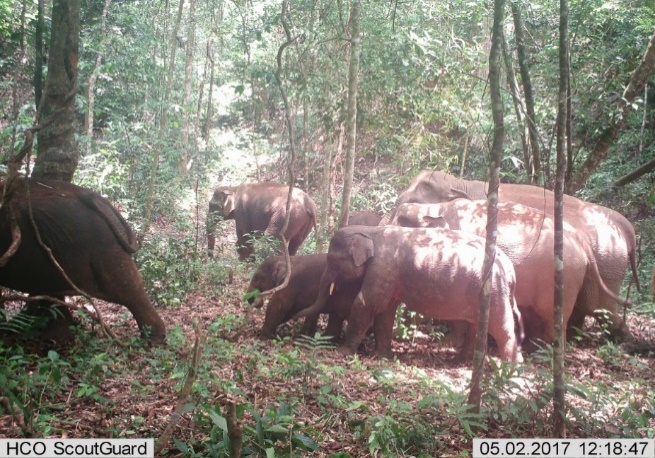

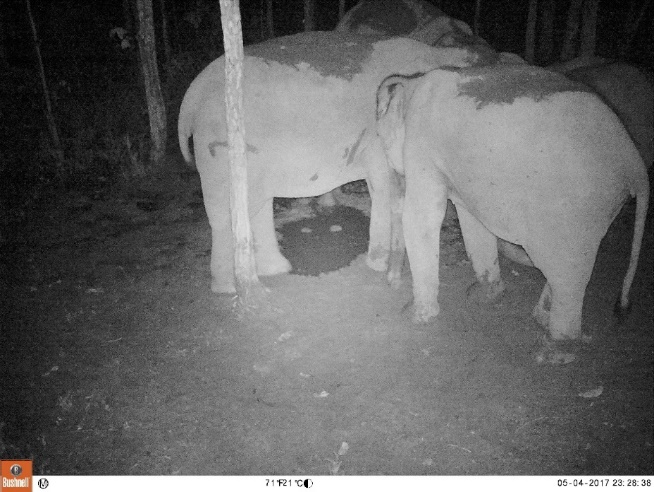


D

C

B

A


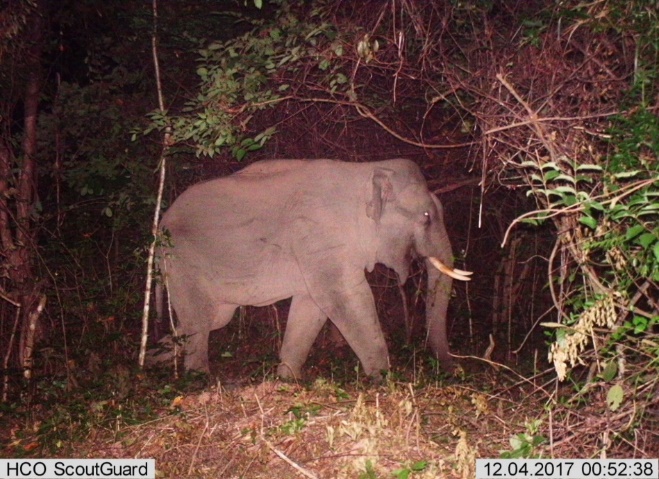

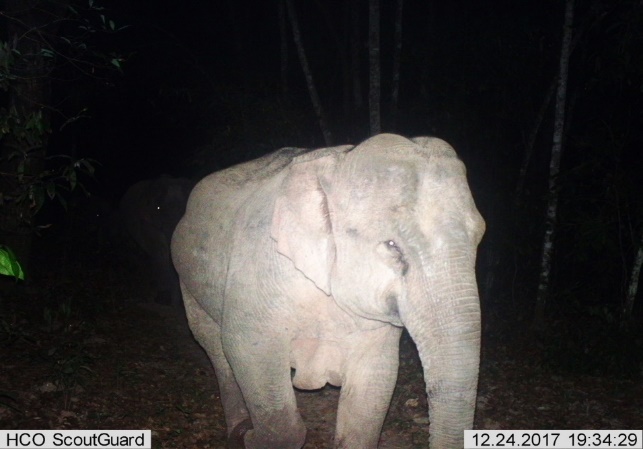


F

E

**Fig. 1** Wild Asian elephant age classes: calf (A), juvenile (B), sub-adult male (C), sub-adult female (D), adult male (E) and adult female (F) in the Phu Khieo Wildlife Sanctuary, Thailand

**2. Wild Elephant Sex Identifications**

Tusks

Male and female wild Asian elephants are different in morphology. First, tusk: only some male wild Asian elephants have tusks (Fig. 2A), while female wild Asian elephants do not have tusks (Fig. 2B), Some male and female wild Asian elephants do not have tusks (Fig. 3A) but some male and females have tushes (Fig. 3B).

Body size

The body size of the adult male wild Asian elephant is bigger than the adult female but there are difficulties in identifying the sub-adult age class. For adult females, their breasts are located between their front legs and wild Asian elephant male’s back is V shaped while the female’s is U shaped.


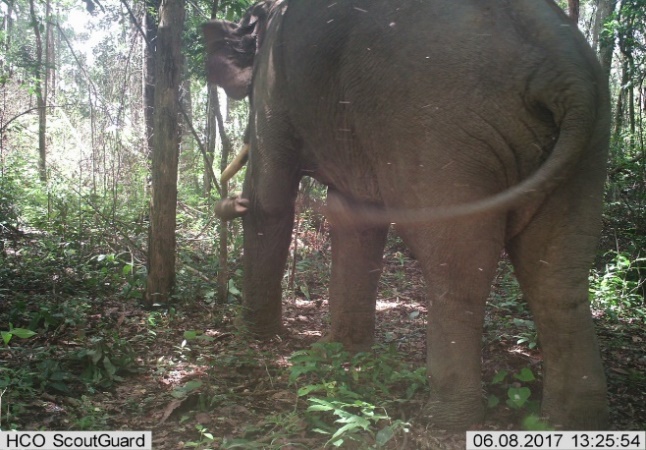

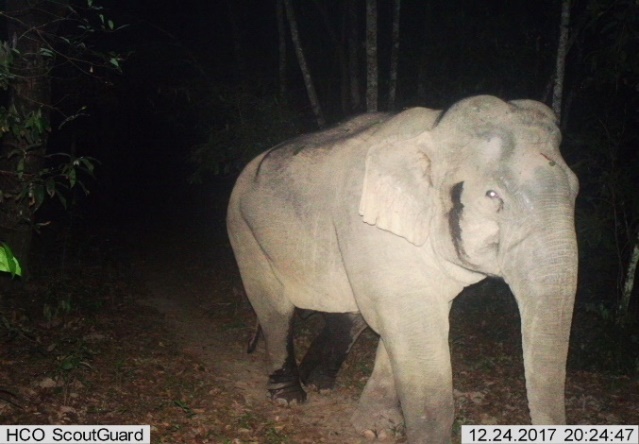


B

A

**Fig. 2** Wild Asian elephant adult male with tusk (A) and without tusk in the field (B).


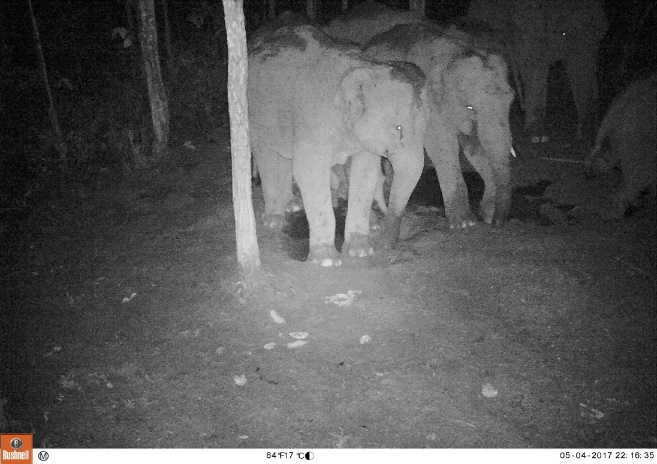

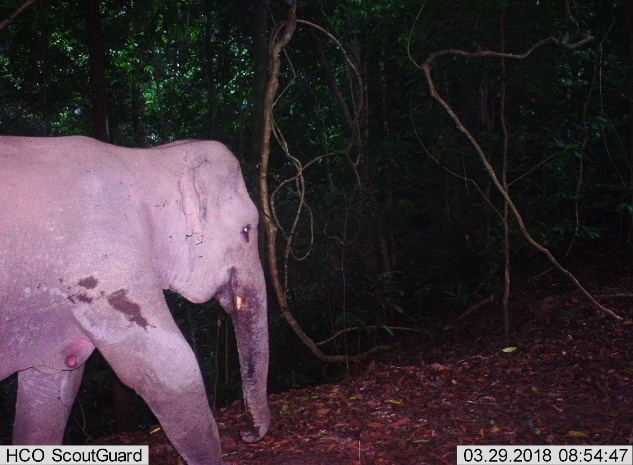


B

A

**Fig. 3** Wild Asian elephant adult female without tushes (A) and with tushes (B) in the field
